# Real-Time 3-Dimensional Echocardiographic Assessment of Ventricular Volume, Mass, and Function in Human Fetuses

**DOI:** 10.1371/journal.pone.0058494

**Published:** 2013-03-14

**Authors:** Minjuan Zheng, Micheal Schaal, Yan Chen, Xiaokui Li, Weihui Shentu, Pengyuan Zhang, Muhammad Ashraf, Shuping Ge, David J. Sahn

**Affiliations:** 1 Pediatric Cardiology, Oregon Health and Science University, Portland, Oregon, United States of America; 2 Department of Ultrasound, Xijing Hospital/Fourth Military Medical University, Xi’an, China; 3 Department of Oncology, Xijing Hospital/Fourth Military Medical University, Xi’an, China; 4 Section of Cardiology, St. Christopher's Hospital for Children/Drexel University College of Medicine, Philadelphia, Pennsylvania, United States of America; Mayo Clinic College of Medicine, United States of America

## Abstract

**Objectives:**

We sought to determine the feasibility and reproducibility of real-time 3-dimensional echocardiography (RT3DE) for evaluation of cardiac volume, mass, and function and to characterize maturational changes of these measurements in human fetuses.

**Methods:**

Eighty pregnant women in the 2^nd^ and 3^rd^ trimesters (59 with normal fetuses and 21 with fetuses with congenital heart disease [CHD]) were enrolled. We acquired RT3DE images using a matrix-array transducer. RT3DE measurements of volume, mass, stroke volume (SV), combined cardiac output (CCO), and ejection fraction (EF) were obtained. Images were scored and analyzed by two blinded independent observers. Inter- and intraobserver variabilities and correlations between fetal cardiac indices and gestational age were determined.

**Results:**

Fifty-two of 59 normal data sets (88%) and 9 of 21 CHD data sets (43%) were feasible for analysis. In normal fetuses, the right ventricle (RV) is larger than the left ventricle (LV) (*P*<0.05), but no difference exists between the LV and RV in mass, SV, CO, and CO/CCO. The EFs for the LV and RV were diminished; the RVSV/LVSV was reduced in CHD fetuses compared with normal fetuses (*P*<0.05). Fetal ventricular volumes, mass, SV, and CCO fit best into exponential curves with gestational age, but LVEF, RVEF, and RVSV/LVSV remain relatively constant.

**Conclusions:**

RT3DE is feasible and reproducible for assessment of LV and RV volume, mass, and function, especially in normal fetuses. Gestational growth of these measures, except for EF, is exponential in normal and CHD fetuses. CHD fetuses exhibit diminished LV and RV EFs.

## Introduction

Ventricular volumetrics are crucial measures for evaluating fetal cardiovascular maturational growth, especially for fetuses with cardiac and extracardiac defects. Accurate and reliable methods for assessing ventricular volumes, mass, stroke volume (SV), cardiac output (CO), and ejection fraction (EF) will provide information to guide medical and surgical management of fetuses with structural and functional abnormalities. Conventional quantitative assessments of these indices in fetuses using 2-dimensional echocardiography (2DE) and pulse-wave Doppler have intrinsic technical limitations in terms of accuracy and have not gained wide acceptance [1]. A spatiotemporal image correlation (STIC) technique and virtual organ computer-aided analysis (VOCAL) have recently been used to assess fetal cardiac volume [2]–[6]. Although the results of these studies have validated the feasibility and accuracy of this technique, it is an indirect motion-gated offline scanning mode rather than a real-time 3-D echocardiography (RT3DE) technique [7].

Technological development in matrix-array and RT3DE allows acquisition of full-volume data sets within seconds in children and adults [8]. To date, although data reported that RT3DE based on matrix probe allows examination of fetal structures from multiple perspectives in real time, which is helpful to confirm normal cardiac structure and detect congenital anomalies [9]–[13], measurements of fetal ventricular volumes, mass, SV, combined cardiac output (CCO), and EF have not been systematically evaluated using matrix-array RT3DE. In addition, the changing roles of the right and left ventricles during fetal growth and development warrant further investigation using a more reliable method. Therefore, our aims were to (1) evaluate the feasibility and reproducibility of RT3DE for measuring ventricular indices in fetuses with normal and abnormal cardiac structures; and (2) evaluate the roles and performance of the left ventricle (LV) and right ventricle (RV) during the second and third trimesters.

## Methods

### Study Subjects

A total of 80 consecutive pregnant women using the perinatal services of the Oregon Health & Science University were enrolled in this study. Written informed consent form was provided by each participant before study. Consent procedure and research protocol were approved by the institutional review board of Oregon Health & Science University. Participants included women with normal fetuses (n = 59; age 18–42 years; fetal gestational age 16.7–34.6 weeks, mean 24.6±5.6 weeks) and women with fetuses with CHD (n = 21; age 20–35 years; gestational age 21.1–35.1 weeks, mean 27.5±4.8 weeks).

In the group with normal fetuses, the indications for ultrasound were confirmation of gestational age and fetal growth, advanced maternal age, abnormal screening test results, previous obstetric complications, family history of congenital heart anomalies, and suspicion of cardiac or extracardiac anomalies. All of the pregnancies met the following criteria: no maternal or fetal complications and normal results on a fetal echocardiogram. The indications for ultrasound in the group with fetuses with CHD are shown in Table 1. Routine obstetric ultrasound scans were followed by 3DE scans. According to the protocol, RT3DE acquisition time was limited to 15 minutes. Women with multiple fetuses were excluded. Postnatal confirmation of CHD was obtained by neonatal echocardiography, cardiac catheterization, or autopsy.

**Table 1:** Characteristics of the congenital heart defects examined by RT3DE.

Disease	Gestational Age(weeks)	No. of fetus	RT3DE information Quality	
			Not measurable*	Useful†
Hypoplastic left heart syndrome	31∼35	4	4	0
Tetralogy of Fallot(TOF)	30∼35	2	1	1
Situs inversus,dextrocardia,complete transposition of the great vessels(TGA)	21∼24	2	2	0
Secundum atrial septum defect(ASD)	23∼32	4	1	3
Ventricular septum defect(VSD)	24∼30	4	0	4
Double outlet right ventricle, TGA	20∼23	2	2	0
Tachycardia,VSD,atrial septal aneurysm	21	1	0	1
Pulmonary atresia with a hypoplastic right ventricle	24	1	1	0
Truncus arteriosus	21	1	1	0
Total cases		21	12	9

* information derived from RT3DE was unclear for diagnosis.

† information derived from RT3DE was useful and obligatory for the diagnosis.

### Image Acquisition

The RT3DE data sets were obtained using a 2- to 4-MHz matrix array X4 transducer and 3DE ultrasound system (HP SONOS 7500, Philips Medical Systems, Andover, MA). The resolution of this system is approximately 0.7×0.7×0.5 mm to 1.2×1.2×0.8 mm as measured by voxel size. 3D data were acquired by Sahn DJ, and Li X and analyzed by Zheng M, Schaal M. When typical four-chamber views were shown, with least fetal movement and with abdomen upwords, it was the best position for fetal heart 3D data acquisition. The image or region of interest was optimized for gain, compression, scanning depth, image width, angles, transmission focus, and line density. We used an external signal generator that was timed to be in synchrony with the observed fetal heart beats on the real time 2D echocardiography, which was used as a trigger for the 3D acquisition. Acquisitions were performed during fetal quiescence with multiple complete cardiac cycles. Each acquisition speed varied from approximately 5 s to 7 s. An average of 5 to 7 loops were obtained and later transferred to a workstation for offline analysis.

### RT3DE Data Analysis

#### Image quality assessment

All the RT3DE data were assessed by scores. The image quality of each data set was assigned to the following score criterion: 1′: unacceptable-image quality was not measurable; 2′: poor-image was judged as measurable but was suboptimal with artifacts; 3′: acceptable-image was clear, measurable and diagnostic; 4′: good-image was good, easily measurable and artifact was rarely present; 5′: excellent-image was extremely clear with all sharp borders for confident diagnosis and measurement.

#### LV analysis

**Figure 1:**
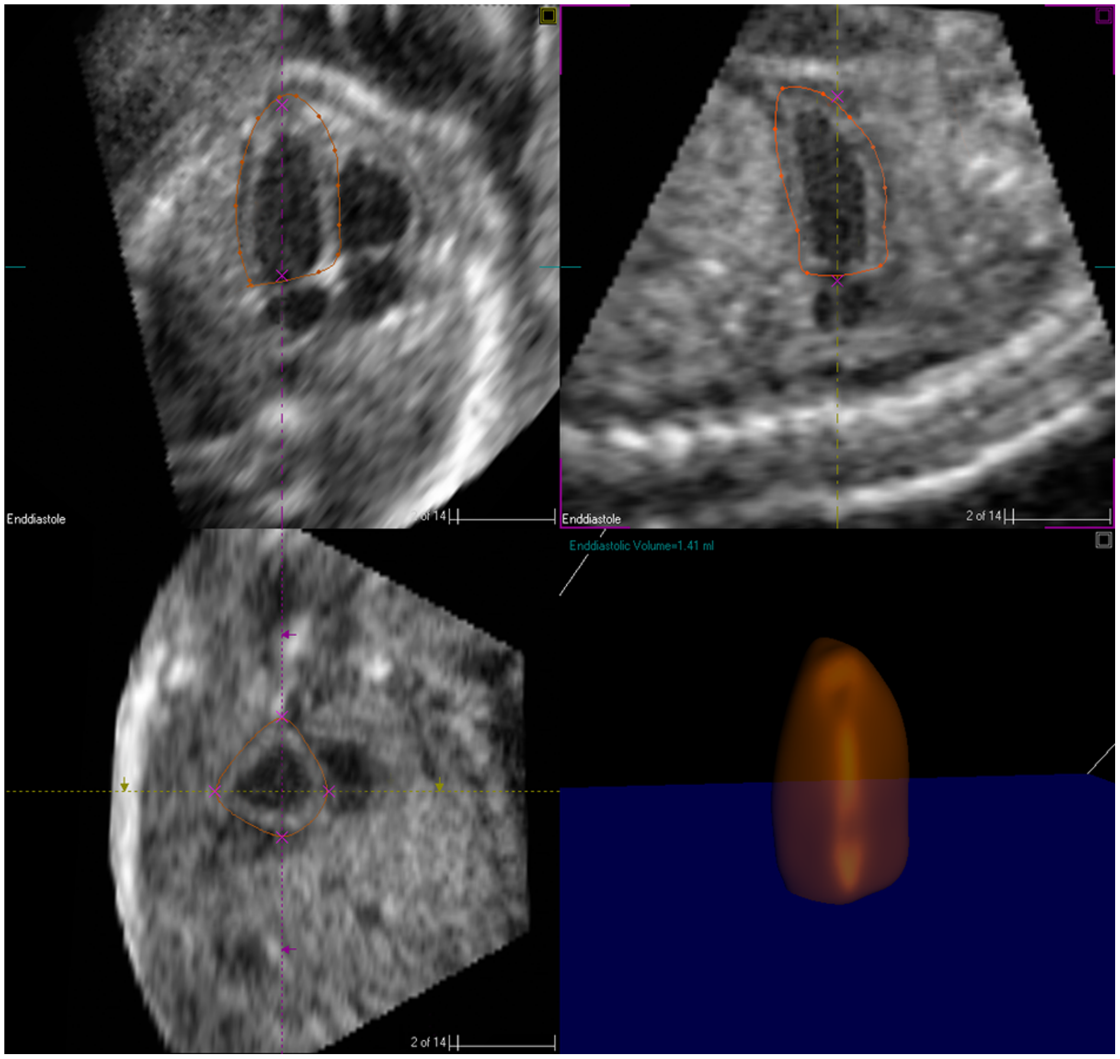
RT3DE image analysis for LV volumes and mass based on 3 orthogonal planes. Upper left, 4-chamber view with easily identifiable morphological markers. Upper right,2-chamber view planes, Lower left, short-axis view of both ventricles, lower right, calculated LV volume and shape display.

LV 3D data were analyzed by a 4-plane algorithm (4D LV-Analysis software package, Research-Arena, TomTec, Munich, Germany) (Figure 1). First, the data set was oriented, adjusted, and displayed in four views, including three long-axis views (apical 4-, 3-, and 2-chamber views) and an orthogonal short-axis view. Second, an end diastolic (ED) frame was chosen as the largest chamber size and an end systolic (ES) frame, as the smallest chamber size. Third, initial manual tracing of the endocardial and epicardial contours was performed using the 4-, 3- and 2-chamber views during end diastole and end systole and adjustment was made with reference to the short-axis view and cine loops. Finally, semiautomatic endocardial and epicardial tracing was undertaken on both end diastole and end systole, and 4-dimensional LV indices were calculated. A minimum of 3 loops were traced to derive the average for each data set.

#### RV analysis

**Figure 2:**
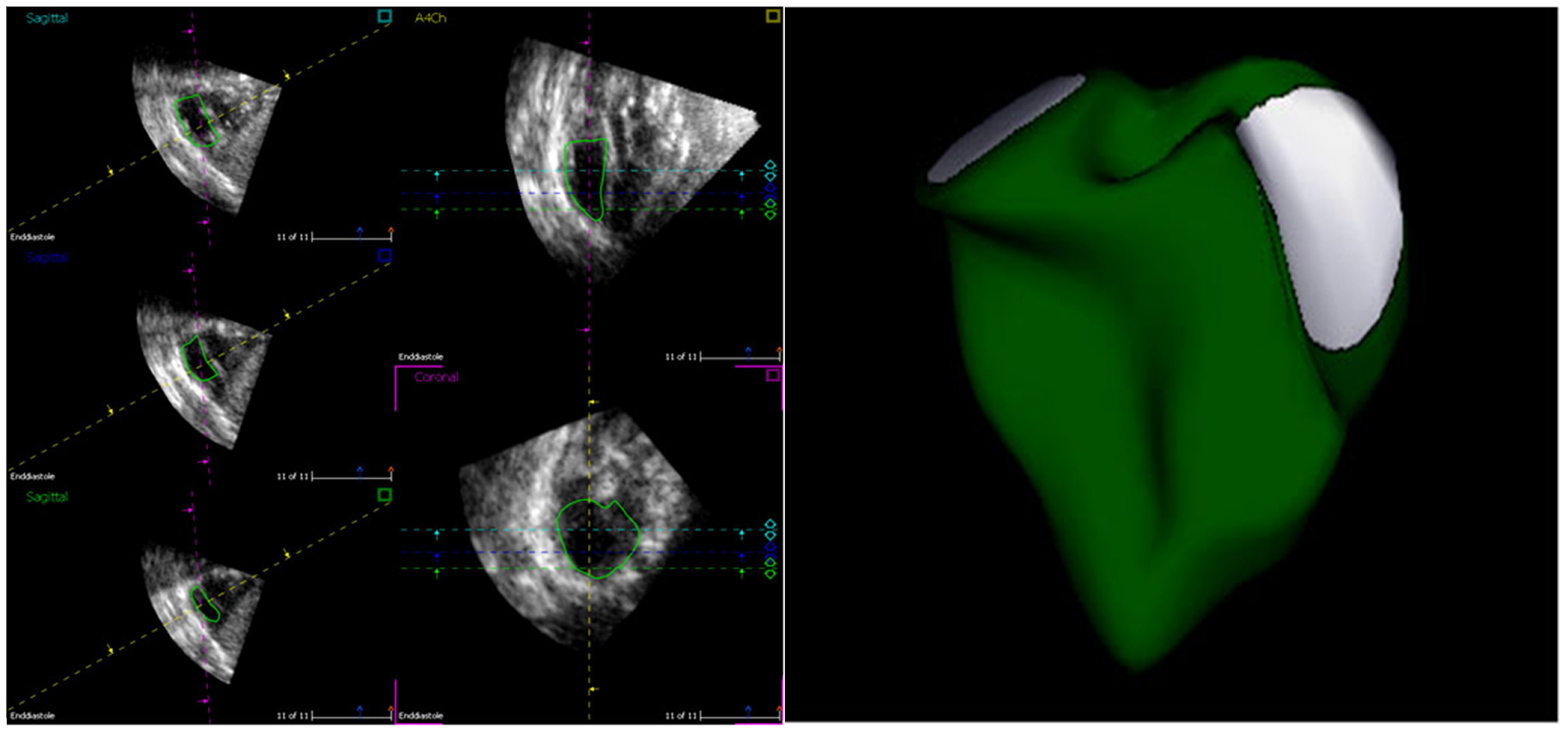
RV volume analysis by RT3DE image package. Left, the 3D data displayed in sagittal, 4-chamber, and coronal views, right, displayed RV volume and shape.

RV 3D data were analyzed in a similar fashion using a 4D RV-Function software package (Research-Arena, TomTec, Munich, Germany). By contrast, the landmarks were set using the center of the tricuspid valve, mitral valve, RV, and LV. The 3D data were oriented, adjusted, and displayed in sagittal, 4-chamber, and coronal views (Figure 2). Again, initial manual tracing of the endocardial and epicardial contours was performed using these images. Finally, semiautomatic endocardial and epicardial tracing was undertaken on end diastole and end systole, and 4-dimensional RV indices were calculated.

#### LV and RV calculations

Stroke volume (SV) was calculated as SV = EDV−ESV. Ejection fraction (EF) was calculated as EF = (EDV−ESV)/EDV. Cardiac output (CO) was calculated as CO = SV × heart rate (HR). Combined cardiac output (CCO) was calculated as CCO = LV CO+RV CO. Myocardial mass was calculated as myocardial volume × myocardial density (1.05 g/mL) [14], where myocardial volume was the difference between epicardial and endocardial shells of the LV or RV. Papillary muscle and trabeculae were included in the endocardial rim, but the apical RV moderator band was excluded and considered as cavity volume.

### Intra- and Interobserver Variability of RT3DE Measurements

A total of 30 3DE data sets were selected randomly. Tracings and measurements were performed by two blinded experienced observers to determine intra- and interobserver variability. To ascertain intraobserver variability, one observer, who was blinded to the first analysis and measurements, repeated the RT3DE data analysis at a 4-week interval. To determine interobserver variability, a second observer analyzed and measured the RT3DE data independently, blinded to the results of the first observer. Intra- and interobserver variability was expressed as a percentile of the differences (difference between the measurements/mean of the measurements ×100%).

### Statistical Analysis

Continuous variables were shown as the mean ± the standard deviation. Interobserver agreement of image scores was assessed using kappa statistics, with a kappa value greater than 0.8 and 0.61 to 0.80 representing good and substantial agreement, respectively [15]. The Pearson chi-square test was used to check for independence between image scores and gestational age. The Bland–Altman analysis was used to examine agreement within or between observers. Correlation and regression analyses were used to determine the relationships between gestational age and ventricular indices derived by RT3DE. The significance of the biases was tested using a 2-tailed *t* test. Levene’s test was used to assess the equality of variance. Statistical significance was defined as *P*<0.05. Statistical analyses were done using the SPSS statistical software package (version 13.0; SPSS Inc, Chicago, IL) and Microsoft Excel 2007 (Microsoft Corporation, Redmond, WA).

## Results

### Feasibility and Reproducibility of RT3DE Fetal Measurements

As shown in Table 2, the feasibility of RT3DE measurements of 80 fetuses (59 normal and 21 CHD) were assessed by an image quality scores system. Of the 59 normal fetuses, 88% (52/59) were suitable for 3DE analysis (n = 52; age 18–42 years; gestational age 16.7–34.6 weeks, mean 23.7±4.2). For fetuses with CHD, 43% (9/21) of the data sets were acceptable for 3D analysis. The characteristics of the CHD fetuses are summarized in Table 1 (n = 9; age 27–35; gestational age 21–35 weeks, mean 27.1±4.9). No adverse event occurred.

**Table 2:** RT3DE image quality scores.

GestationalAge(weeks)	Image Quality Scores					
	1′	2′	3′	4′	5′	total
≥16∼<21	5(6.25%)	2(2.5%)	9(11.3%)	4(5.0%)	0	20
≥21∼<29	8(10.0%)	4(5.0%)	12(15.0%)	7(8.75%)	14(17.5%)	45
≥29∼<40	6(7.5%)	2(2.5%)	3(3.75%)	3(3.75%)	1(1.25%)	15
Total	19	61				80

The 3D image quality was assessed as follows:

1′: *unacceptable*-image quality was not measurable;

2′: *poor*-image was judged as measurable but was suboptimal with artifacts;

3′: *acceptable*-image was clear, measurable and diagnostic;

4′: *good*-image was good, easily measurable and artifact was rarely present;

5′: *excellent*-image was extremely clear with all sharp borders for confident diagnosis and measurement.

When image quality was assessed, a chi-square test of image scores and gestational age showed χ^2^ = 12.86, *P* = 0.12. The kappa statistics indicated interobserver agreement for 3D image quality scores (κ value = 0.76; *P*<0.001). The inter- and intraobserver variabilities for RT3DE measurements were 1.3±3.9% and 0.7±6.1% for volumes, and 1.0±3.6% and 2.5±5.4% for ventricular mass.

### RT3DE Assessment of Fetal Ventricular Volume, Mass, and Function

RV and LV cardiac indices were obtained from each adequate image set. The time necessary for analyzing both LV and RV volumes and mass after image acquisition was from 7 to 26 minutes (average 12.9±7.8 minutes). The mean values of EDV, ESV, and mass obtained with RT3DE are shown in Table 3. There was no significant difference in EDV, ESV, and mass between the normal and CHD groups (*P*>0.05). However, RVEDV and RVESV were significantly larger than LVEDV and LVESV in normal fetuses, respectively (*P*<0.05).

**Table 3:** Mean Ventricular Volumes, Mass by RT3DE in Normal and CHD Fetuses.

	Age	GA	HR	LVEDV,ml	LVESV,ml	RVEDV,ml	RVESV,ml	LVmass,gm	RVmass,gm
Normal(n = 52)	28.6±5.6	23.7±4.2	136.7±5.4	1.88±1.56*	0.90±0.78*	2.04±1.50	1.05±0.80	1.77±1.45	1.79±1.19
CHD (n = 9)	30.0±2.6	27.1±4.9	132.3±8.0	1.89±0.95	0.80±0.49	1.89±0.93	0.80±0.43	1.63±1.07	1.54±0.97

* P<0.05: compared with right ventricle; P>0.05: compared normal group with CHD group in all indices.

GA: gestational age, HR: heart rate.

**Figure 3:**
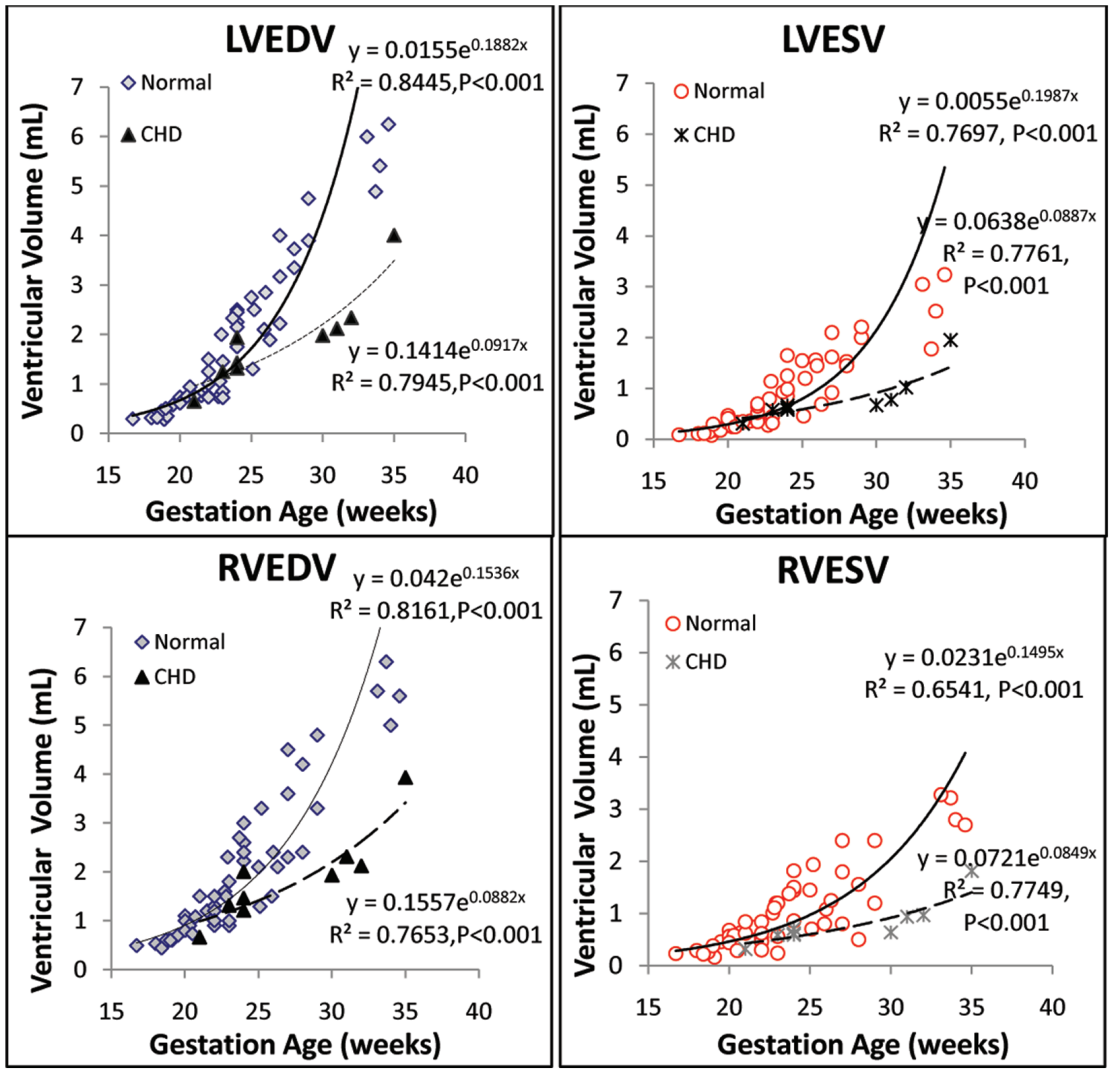
Gestational growth of LV and RV volumes in normal and CHD fetuses.

**Figure 4:**
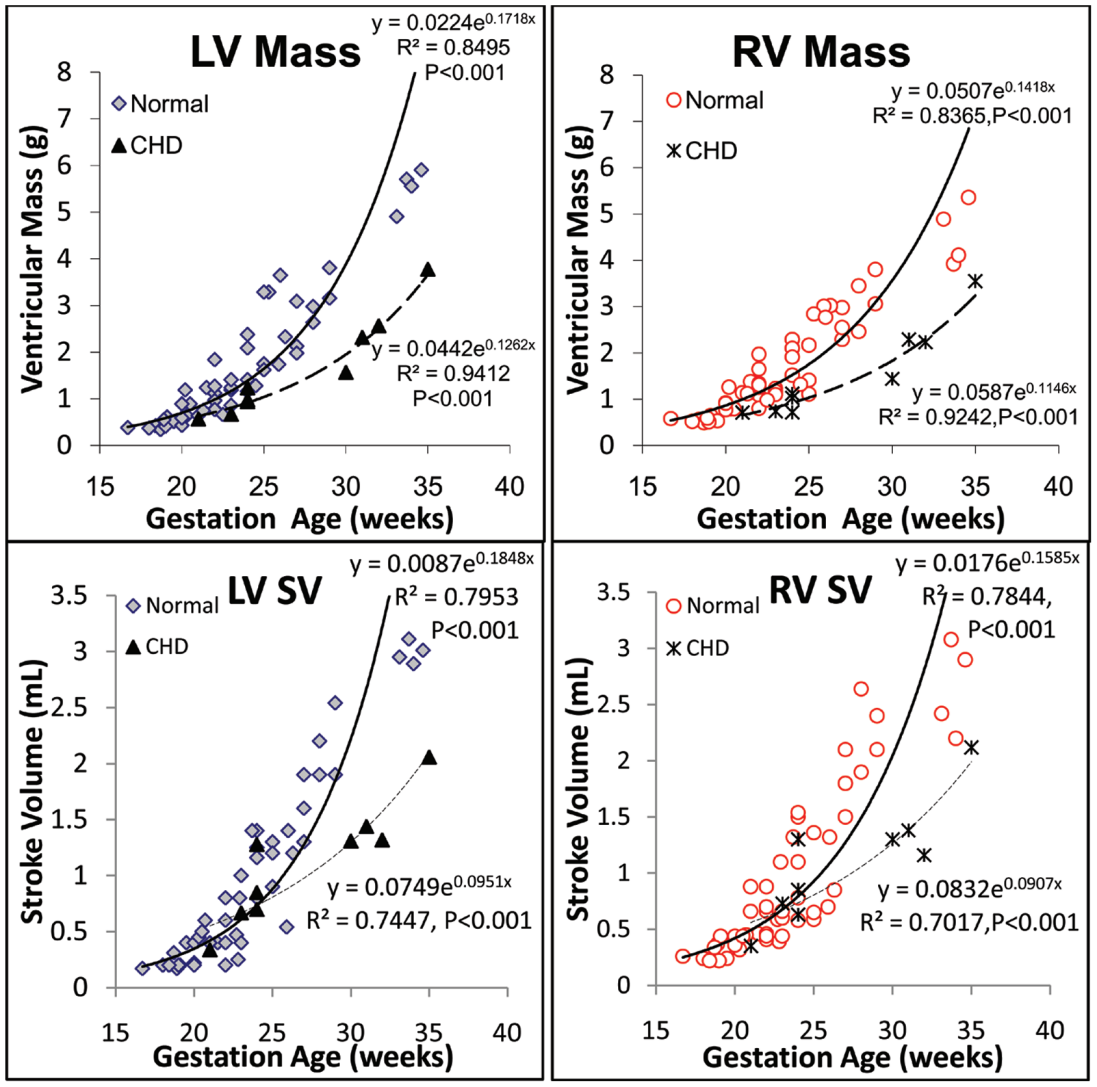
Growth of LV and RV mass and stroke volume with gestational age in normal and CHD fetuses.

**Figure 5:**
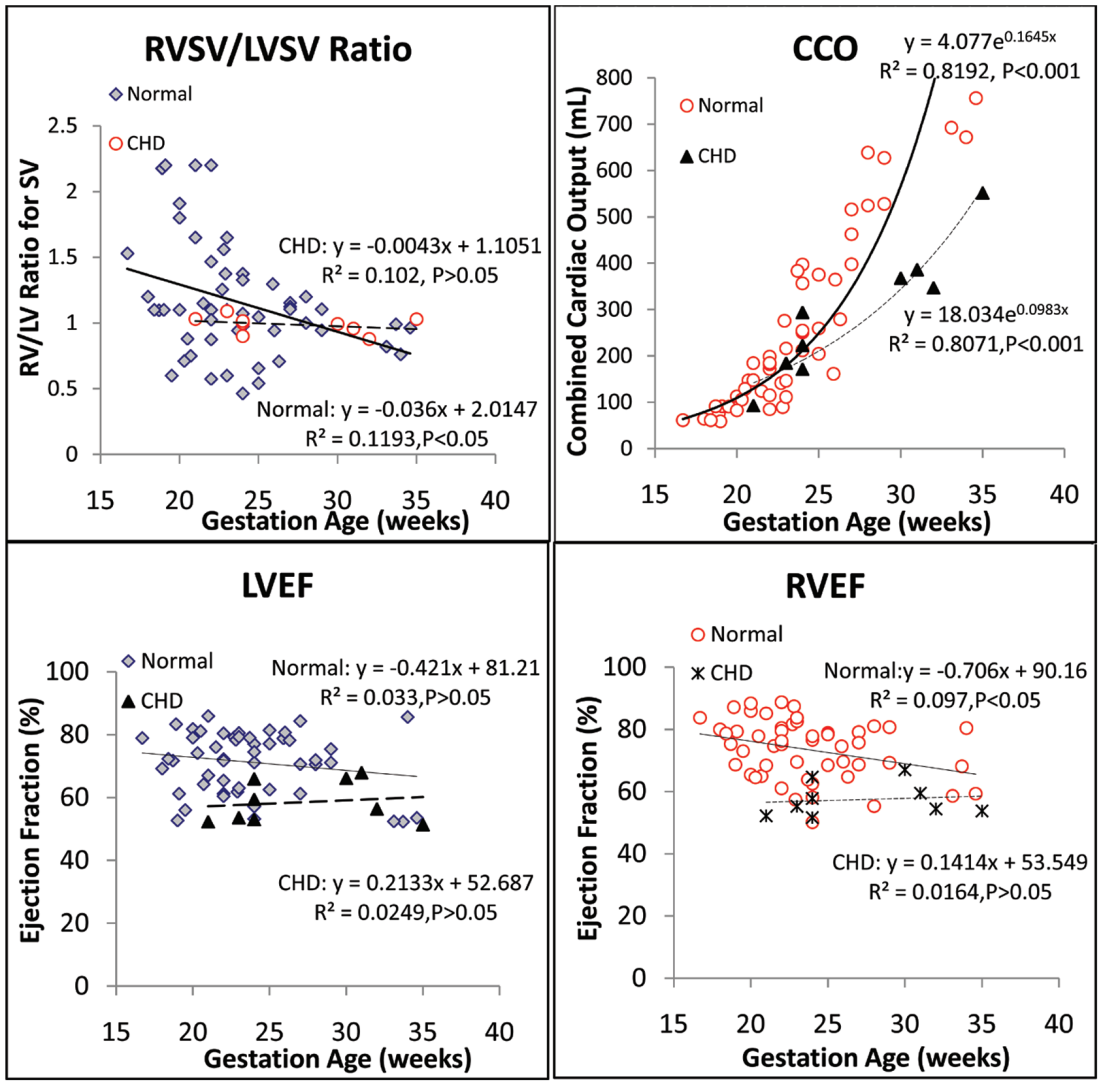
Gestational growth and maturation of RV to LV SV ratio, LV and RV EFs, and CCO.

When ventricular volumes, masses, SV, and CCO in both normal and CHD groups were correlated with gestational age (Figures 3, 4, 5), the best fits were curvilinear, that is, exponential with gestational age. Although we found no statistically significant difference between the normal and CHD groups, the two groups exhibited different regressions.

Right and left ventricular SV, CO, CCO, and EF for the normal and CHD groups are shown in Table 4. No difference was seen in SV and CO between the LV and RV in both normal and CHD groups, nor was any difference noted in the LVCO/CCO and RVCO/CCO. Furthermore, although the results trended toward increased RV and LV SV, CO, and CCO in the group with CHD, the difference was statistically insignificant. However, we noted a significant decrease in RVEF, LVEF, and RV/LV SV in the CHD group compared with the normal group (Table 4). In addition, the RVSV/LVSV ratio, LVEF, and RVEF seemed declined with gestational age in the normal group, but only RVSV/LVSV ratio and RVEF showed a significant negative correlation (Figure 5).

**Table 4:** Ventricular SV, CO, CCO and EF by RT3DE in Normal and CHD Fetuses.

	LVSV,ml	RVSV,ml	LVCO,ml	RVCO,ml	CCO,ml	LVCO/CCO	RVCO/CCO	LVEF,%	RVEF,%	RV/LV SV
Normal(n = 52)	0.98±0.82	0.99±0.77	131.2±107.3	133.1±100.7	264.3±205.1	0.48±0.09	0.52±0.09	71.3±9.8*	73.5±9.5*	1.16±0.44*
CHD (n = 9)	1.10±0.51	1.09±0.52	146.7±70.0	144.3±69.8	291.1±139.4	0.50±0.01	0.50±0.02	58.5±6.6	57.4±5.4	0.99±0.07

* P<0.05, compared with CHD group. P>0.05, compared left ventricle with right ventricle in all indices.

## Discussion

### Real-time 3-Dimensional Fetal Echocardiography

Previous assessment of fetal ventricular volume growth and functional maturation has largely been based on invasive means in instrumented animals and M-mode or 2D studies in humans [16], [17]. Whereas these measurements have been validated in adult, children, and fetuses, evidence is growing that these measurements have limited accuracy and reproducibility compared with current real-time 3D echocardiography in both adults and children. M-mode echocardiography is highly dependent on the incident ultrasound beam and geometric assumptions of the ventricles. It has limited accuracy and repeatability [18]. As many as 40% of normal fetuses can have falsely increased cardiac dimensions on random 2D measurements compared with phase-specific M-mode dimensions [19]. It has also been shown that 2D-based methods provide reasonable estimates of ventricular indices for the LV but are problematic for the RV [20], [21]. In an adult study, the 2D-based echocardiographic calculations overestimated RV volumes by as much as 30% to 35% [14]. These potential measurement errors may be more problematic when the small size of the ventricles and the small absolute functional changes are evaluated in human fetuses with normal and congenital heart defects.

Three-dimensional echocardiography has become available for prenatal diagnosis of heart defects. Quantitative 3D echocardiography has also been used to determine ventricular volume, mass, and function in both children and adults [22]–[24]. Most 3DE fetal studies have used the spatiotemporal image correlation (STIC) technique [2]–[7]. The STIC technique is based on motion-gated offline reconstruction of consecutive subvolumes during a cardiac cycle. The automated volume acquisition is made possible because the array in the transducer performs a slow single sweep, recording a single 3D data set consisting of many 2D frames, one behind the other, then combining frames from the identical phase in the cycle from consecutive slices. When the cardiac abnormality involves dyssynchrony between ventricles, this method may lead to inaccurate estimation of maximum and minimum volume, such as EDV and ESV.

In contrast, 3DE with xMatrix transducer technology can facilitate real-time 3D visualization of the fetal heart. In this approach, the transducer is usually built with 3000 active elements to capture data to generate real-time 3D images instead of reconstructing 2D images with STIC technology.

### Feasibility and Reproducibility

Based on the feasibility data obtained in this study, 3D data from 88% normal and 43% CHD fetuses were adequate for analysis. The correlation between the 3D image scores and gestational age showed that the image quality did not change significantly after 16 weeks of gestation, suggestive of the multifactorial contribution to the 3D image quality, probably related to fetal position, maternal acoustic window, and amniotic fluid quantity and clarity, in addition to gestational age. In our study, feasibility was low for 3D data set for CHD fetuses (9/21, 43%). In this cohort, the success rate of fetal RT3DE was related to whether we can get ideal four chamber view. In the 9 measurable cases, 8 cases were with simple CHD and had nearly normal size ventricles (3 ASD, 5 VSD, Table 1). In other 12 cases not suitable for 3D analysis, mostly were complex CHD, including 7 cases with significantly hypoplastic ventricles or large septal defects (4 Hypoplastic left heart syndrome, 1 TOF, 1Pulmonary atresia with a hypoplastic right ventricle, 1Truncus arteriosus), or malpositioned-ventricles and/or with significantly altered ventricular geometry (4 TGA, 2 of them were situs inversus and dextrocardia), which were not suitable for LV and RV analysis algorism and software. The kappa statistics indicated good interobserver agreement for 3D image quality scores (κ value = 0.76; *P*<0.001). In addition, Bland–Altman analyses showed good inter- and intraobserver reproducibility for ventricular volumes and mass.

### Ventricular Volume, Mass, and Function

A number of studies have pursued the use of reconstructive 3D echocardiography and STIC to quantify fetal heart volumes, mass, and function [2]–[6], [25]. Simioni et al established nomograms for fetal SV, CO and EF by STIC modality, finding SV and CO increased exponentially with gestation and EF remained fairly stable through gestation (around 63%) [26]. Our finding is accordant with theirs with exponentially increased SV, CO, and relative stable EF (Figure 4 and 5). The ranges of these variables were wider, which may due to wider gestation age ranges (16.7 to 34.6 weeks compared with 20 to 34 weeks). Messing et al also estimated fetal ventricular mass using STIC with VOCAL inversion mode [27]. LV and RV mass values derived from this method was larger than our data (especially RV mass). Besides systematic variation between STIC VOCAL and RT3DE, this difference may also caused by their larger sample size (106 fetus including normal and abnormal), and with larger ranged of ventricular sizes (including cardiomyopathy cases with enlarged chambers). Like RT3DE, STIC volumes can also be calculated automatically. Rizzo G et al reported good agreement between VOCAL and sonographic automatic volume calculation (sonoAVC) for fetal ventricular volume measurements (intraclass correlation coefficients 0.978 and 0.985 for LV and RV), and the time necessary to measure the SV was significantly shorter with sonoAVC (2.8 versus 11.7 minutes) than with VOCAL [28].

Using real-time 3D echocardiography, we demonstrated an exponential growth pattern for ventricular volumes and mass. When we compared data from the normal group with those from the CHD group, although we noted a trend toward smaller chamber size, mass, SV, CO, and CCO, we found no significant difference in EDV, ESV, mass, SV, CO, or CCO. However, we did find a significant difference in EF and the RV to LV ratio for SV. These findings may be due to the small number of fetuses with CHD relative to the number of normal fetuses. However, it is also plausible that EF and the lesser role RV played as compared with LV were more sensitive to fetal functional compromise and adaptation secondary to CHD.

### Fetal Cardiac Output and Distribution

Much of our current understanding about fetal cardiac output and distribution is derived from studies in fetal sheep. In these studies, fetal CO and distribution were measured using the radionuclide-labeled microsphere method or electromagnetic flow transducers applied around the ascending aorta and the pulmonary trunk [29], [30]. Results from these methods indicated that, in the fetal lamb, the RV ejected approximately two-thirds and the LV ejected one-third of the combined ventricular output. Results from previous human fetal studies using Doppler echocardiographic measurements of the CCO indicated that, although the RV was dominant in the human fetus, the human RV to LV ratio was smaller (1.2–1.5) than that in the fetal lamb (1.8) [30]–[32]. According to our RT3DE data, the RV-to-LV ratio for SV in the normal group was 1.16±0.44, which was different from that for the CHD group (0.99±0.07), yielding values closer to those obtained using Doppler echocardiography than were found in the sheep studies.

In fetal circulation, the LV output is directed into the ascending aorta to supply the upper body, including the brain, whereas the RV output supports the lower body and placenta through the ductus arteriosus. Therefore, the large brain mass of humans compared with that of sheep may explain the higher LV output and the lower ratio between the RV and LV output.

For the gestational EF changes and the RV/LV ratio of SV as linear regression fits, a trend toward a decrease in EF and RV/LV SV with gestational age was observed (Figure 4); again, we found no statistical significance, except for RV/LV SV and RVEF for normal fetuses, which was only a modest correlation (R^2^ = 0.12, *P*<0.05, R^2^ = 0.10, *P*<0.05, respectively) (Figure 5).

### Study Difficulties and Limitations

The study has several limitations: We do not have a gold standard, either ex vivo or in vivo, for validation of RT3DE technology; nor do we have experimental data to corroborate the findings from 3D echocardiography, such as direct comparison with 3D volume and mass by fetal cardiac MRI. Most of the available validation studies derived from adults and children show the superior accuracy and reproducibility of 3D versus 2D or M-mode echocardiography for measurement of volumes, mass, and function. As with 2D studies, the 3D method cannot be used to study fetal hearts before 15 gestational weeks because the volumes may be under the lower limit of image resolution. At a fetal gestational age greater than 37 weeks, one may encounter difficulties stemming from fetal position and calcification of the thoracic cavity. In addition, this study is a prospective study from a single institution. The sample size was limited, especially for the group with CHD, and a subgroup analysis based on the type of CHD was not possible. Another limitation that we did not perform a direct comparisons between RT3DE with STIC technology for assessment of cardiac volumes, since the 2 different techniques would require 2 imaging modalities with 2 separate 3D data acquisition and analysis systems.

Despite these limitations, the results are of interest from a developmental perspective and add new and possibly more quantitative measures for fetal cardiac growth and maturational changes in normal and CHD fetuses.

### Conclusions

RT3DE is feasible and reproducible for assessment of LV and RV volume, mass, and function after 15 weeks of gestation, especially in normal fetuses. Increases during gestation of these measures, except for EF, are exponential in both normal and CHD fetuses. Although RV is the dominant ventricle, the ratio of the RV and LV contribution to the combined CO is lower in humans than in experimental animals. LV and RV EFs are compromised in CHD fetuses compared with normal fetuses and may serve as sensitive measures for evaluation of fetal cardiac function.
